# Effects of Vitamin D on Cardiac Function in Patients With Chronic HF: The VINDICATE Study

**DOI:** 10.1016/j.jacc.2016.03.508

**Published:** 2016-06-07

**Authors:** Klaus K. Witte, Rowena Byrom, John Gierula, Maria F. Paton, Haqeel A. Jamil, Judith E. Lowry, Richard G. Gillott, Sally A. Barnes, Hemant Chumun, Lorraine C. Kearney, John P. Greenwood, Sven Plein, Graham R. Law, Sue Pavitt, Julian H. Barth, Richard M. Cubbon, Mark T. Kearney

**Affiliations:** a Leeds Institute of Cardiovascular and Metabolic Medicine, University of Leeds, Leeds, United Kingdom; b Leeds Teaching Hospitals NHS Trust, Department of Cardiology, Leeds, United Kingdom; c School of Dentistry, University of Leeds, Leeds, United Kingdom; d Leeds Teaching Hospitals NHS Trust, Department of Clinical Biochemistry, Leeds, United Kingdom

**Keywords:** heart failure, left ventricular function, remodeling, vitamin D, 1,25 (OH)2 vitamin D_3_, calcitriol, 25(OH) vitamin D_2_, ergocalciferol, 25 (OH) vitamin D_3_, cholecalciferol, AF, atrial fibrillation, CI, confidence interval, CMR, cardiac magnetic resonance, HF, heart failure, LV, left ventricular, PTH, parathyroid hormone, SR, sinus rhythm

## Abstract

**Background:**

Patients with chronic heart failure (HF) secondary to left ventricular systolic dysfunction (LVSD) are frequently deficient in vitamin D. Low vitamin D levels are associated with a worse prognosis.

**Objectives:**

The VINDICATE (VitamIN D treatIng patients with Chronic heArT failurE) study was undertaken to establish safety and efficacy of high-dose 25 (OH) vitamin D_3_ (cholecalciferol) supplementation in patients with chronic HF due to LVSD.

**Methods:**

We enrolled 229 patients (179 men) with chronic HF due to LVSD and vitamin D deficiency (cholecalciferol <50 nmol/l [<20 ng/ml]). Participants were allocated to 1 year of vitamin D_3_ supplementation (4,000 IU [100 μg] daily) or matching non−calcium-based placebo. The primary endpoint was change in 6-minute walk distance between baseline and 12 months. Secondary endpoints included change in LV ejection fraction at 1 year, and safety measures of renal function and serum calcium concentration assessed every 3 months.

**Results:**

One year of high-dose vitamin D_3_ supplementation did not improve 6-min walk distance at 1 year, but was associated with a significant improvement in cardiac function (LV ejection fraction +6.07% [95% confidence interval (CI): 3.20 to 8.95; p < 0.0001]); and a reversal of LV remodeling (LV end diastolic diameter -2.49 mm [95% CI: -4.09 to -0.90; p = 0.002] and LV end systolic diameter -2.09 mm [95% CI: -4.11 to -0.06 p = 0.043]).

**Conclusions:**

One year of 100 μg daily vitamin D_3_ supplementation does not improve 6-min walk distance but has beneficial effects on LV structure and function in patients on contemporary optimal medical therapy. Further studies are necessary to determine whether these translate to improvements in outcomes. (VitamIN D Treating patIents With Chronic heArT failurE [VINDICATE]; NCT01619891)

Chronic heart failure (HF) secondary to left ventricular (LV) systolic dysfunction (LVSD) is a common condition affecting 5 million individuals in the United States (1) and a similar number in Western Europe (2). While the prognosis of chronic HF has improved substantially during the last 2 decades (3), mortality remains high with 50% of patients dying within 5 years of diagnosis 4, 5.

Patients suffering from cardiovascular disease are frequently deficient in the steroid hormone vitamin D, and vitamin D deficiency has been shown to be associated with the development of chronic HF in a number of studies 6, 7, 8, 9, 10. Approximately 90% of chronic HF patients have hypovitaminosis D (11), even in sunny climates (12). The agent has a range of pleiotropic effects that in the setting of chronic HF may impact on disease severity 13, 14, but despite this, clinical trials examining vitamin D supplementation in chronic HF patients have to date been inconclusive 15, 16.

The aim of the VINDICATE (VitamIN D treatIng patients with Chronic heArT failurE) study was to describe the safety and efficacy of long-term, high-dose vitamin D_3_ supplementation on submaximal exercise capacity and cardiac function in patients with chronic HF due to LVSD.

## Methods

### Study population

VINDICATE was a randomized placebo-controlled double-blind trial of vitamin D supplementation in vitamin D−deficient chronic HF patients on optimal medical therapy. Patients were eligible if they had stable (>3 months) New York Heart Association functional class II or III symptoms, a left ventricular ejection fraction (LVEF) ≤45% on maximally tolerated medical therapy (>3 months) and a 25(OH) vitamin D level of <50 nmol/l (<20 ng/ml).

Patients were ineligible if they were taking or had taken calcium or other vitamin supplements in the last 3 months; if their chronic HF was due to untreated valvular heart disease, anemia or thyrotoxicosis; if they had existing indications for vitamin D supplementation (e.g., previous osteoporotic fracture or symptoms of osteomalacia); if they had a history of primary hyperparathyroidism, sarcoidosis, tuberculosis or lymphoma, a cholecalciferol concentration at the time of screening >50 nmol/l (20 ng/ml); or if there was significant renal dysfunction (estimated glomerular filtration rate <30 ml/min).

### Allocation and intervention

Patients enrolled into VINDICATE were allocated in blocks of 20 using minimization balancing for etiology of chronic HF (ischemic/non-ischemic), diabetes mellitus, sex, chronic obstructive pulmonary disease (requiring use of regular bronchodilators), and ethnic origin (Caucasian/non-Caucasian). Each participant was asked to take 2 tablets per day providing either a total of 100 μg cholecalciferol (4,000 IU daily) or placebo (Cultech, Port Talbot, Wales, United Kingdom).

The supplement and dose were chosen based upon guidelines for studies of vitamin D supplementation (17). These guidelines suggest that studies should: 1) aim to replace physiological requirements, supplementing between 75 and 250 μg/day; 2) last at least 9 months; 3) supplement with vitamin D_3_ (not D_2_); 4) assay supplements for potency; 5) include a regular serum measurement of 25(OH)D_3_ levels; and 6) aim to achieve serum levels in patients on active therapy between 100 and 160 nmol/l (40 ng/ml to 64 ng/ml). Also, on the basis of recent data demonstrating the adverse effect of hyperparathyroidism in chronic HF (18), we chose a dose likely to suppress parathyroid hormone (PTH) release. Our proof of concept study, using the same inclusion and exclusion criteria and protocol as VINDICATE, had previously demonstrated the efficacy of 4,000 IU daily to achieve positive remodeling with significant reductions in left ventricular end-diastolic volume (LVEDV), left ventricular end-systolic volume (LVESV), and left ventricular end-diastolic dimension (LVEDD). The consort diagram and results from this study are presented in online supplementary datasets (Online Figure 1, Online Tables 1 and 2). A simple linear model-based trend test from this study demonstrated a significant decrease in PTH over the year (p = 0.0095) in those allocated vitamin D, with no such trend in patients allocated to the placebo arm (p = 0.977) (Online Figure 2) (19).

### Outcome variables

The pre-specified primary endpoint in VINDICATE was the difference in change in 6-min walk test distance (6MWT) (baseline to 12 months) between the 2 groups. Key pre-specified secondary endpoints included cardiac structure and function, and safety endpoints of serum calcium concentration, renal function, and vitamin D levels. Hypervitaminosis D was defined as 25(OH)D_3_ >200 nmol/l (80 ng/ml), and hypercalcemia as >2.6 nmol/l (10.4 mg/dl).

### Study procedures

At baseline each patient performed a 6MWT according to standard criteria (20). Each patient also underwent echocardiography and blood sampling for serum calcium, serum creatinine, vitamin D, and PTH levels. Patients were also invited to undergo cardiac magnetic resonance (CMR) imaging to measure LV volumes. Subsequent visits took place at 3, 6, 9, and 12 months and blood draws were repeated at each visit for safety data.

### Serum biochemistry

Serum 25(OH)D_2_ and 25(OH)D_3_ were analyzed by tandem mass spectrometry. Samples were prepared using a protein precipitation reagent containing deuterated cholecalciferol. The supernatant was analyzed on an API5000 LC-MS/MS (AB SCIEX, Warrington, United Kingdom) in atmospheric-pressure chemical ionization mode. The inter-assay coefficient of variability was <10% at all concentrations ranging from 12 nmol/l to 159 nmol/l (4.8 ng/ml to 63.7 ng/ml). Ergocalciferol and cholecalciferol concentrations were summed and reported as total 25(OH)D. We defined deficiency and insufficiency of vitamin D concentrations as <50 nmol/l (20 ng/ml) and <75 nmol/l (30 ng/ml), respectively 21, 22. We also measured serum calcium, creatinine, and PTH (Siemens Advia and Centaur, Siemens Healthcare Diagnostics, Camberley, United Kingdom). To confirm effective conversion of the supplement, we also measured 1,25(OH)D_3_ by radioimmunoassay (IDS, Boldon, United Kingdom) at baseline and 12 months.

### Echocardiography

Echocardiography was performed on all patients at baseline and LV function was assessed according to European Society of Cardiology criteria using Simpson’s biplane measure to determine LVEF (23). Echocardiography was repeated at 12 months. Echocardiograms at both time points were analyzed offline at the end of the study by 2 senior echocardiographers blinded to patient treatment.

### CMR imaging

CMR studies were performed on dedicated 1.5-T or 3-T CMR systems (Philips Healthcare, Best, the Netherlands). The same system was used for baseline and follow-up studies (at 12 months) of individual patients. A multislice multiphase data set covering the entire left ventricle in 10 to 12 short axis slices was acquired using a validated 2-dimensional balanced steady-state free precession pulse sequence (TR 2.8 ms, TE 1.4 ms, flip angle 55°, spatial resolution 2.0 mm × 2.0 mm × 10 mm, no interslice gap, 30 phases/cardiac cycle, 1 slice per breath-hold). Offline analysis by an experienced CMR observer using QMASS V7.0 software (Medis, Leiden, the Netherlands) blinded to study allocation derived end-diastolic and end-systolic LV volumes and LVEF.

### Sample size

VINDICATE was powered to provide information on the patient-oriented outcome of 6MWT. A trial of iron supplementation in a similar patient group had demonstrated that improvements of 30 m to 40 m could be expected with this type of intervention (24). We assumed, based upon our preliminary data from a pilot study (19), that there would be a change between the 2 groups at 12 months of 30 m. The SD of change in 6MWT was estimated from these data; the upper limit of the 80% confidence interval (CI) (estimated using bootstrapping) was used in these calculations to allow for the small sample size in the proof of concept. This determined that 210 patients were required to have 90% power to show a difference in change in 6MWT of 28 m or more with 5% significance (SD = 62). We aimed to recruit 230 patients (115 per group) to allow for ∼10% dropout.

### Statistical analysis

Differences in baseline variables between allocations were tested using Student *t* tests (continuous data) or the chi-square test (categorical data). The analysis of primacy for the main efficacy endpoints was based on analysis of covariance linear models relating differences in the final walk distance and imaging variables by treatment allocation, adjusting for baselines values and reported with 95% CIs (25). All significance tests were 2-sided and called significant at the 5% level. All analyses were conducted in Stata (version 14, StataCorp., College Station, Texas).

### Ethical and safety considerations

A single unblinded observer with no involvement in the patients’ care or study follow-up (J.H.B.) reviewed each vitamin D result at each time point for safety. An agreed operating procedure for any subject who developed a serum vitamin D concentration >200 nmol/l (80 ng/ml) involved reducing the dose of treatment from 2 to 1 tablets per day to maintain patient blinding.

## Results

**Figure 1:**
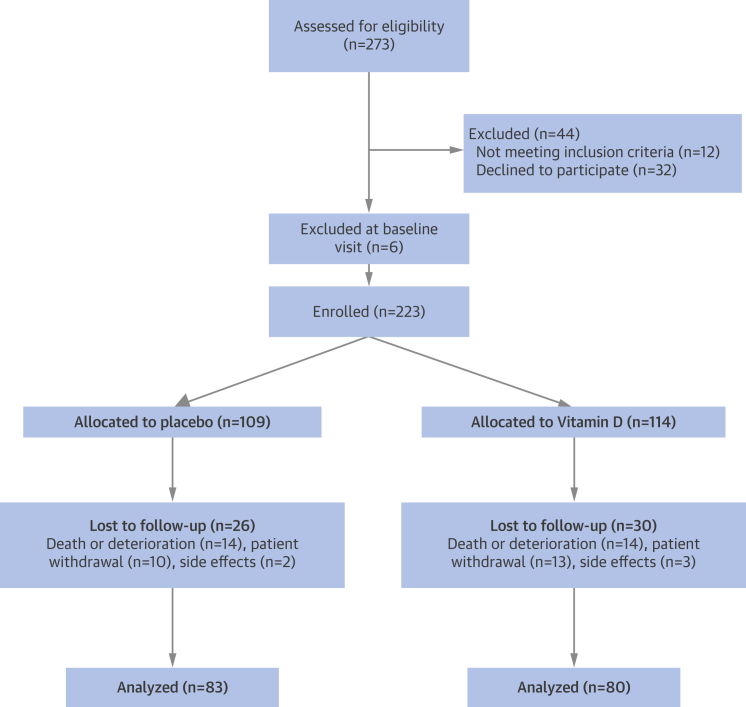
Consort Diagram Demonstrating Patient Enrollment and Disposition for VINDICATE VINDICATE = VitamIN D treatIng patients with Chronic heArT failurE.

We enrolled 229 patients into VINDICATE. Six patients were found to be ineligible at the baseline visit, leaving 223 patients randomized to treatment. Figure 1 describes patient recruitment and loss to follow-up. A total of 163 patients completed the study. Baseline characteristics divided by treatment allocation are shown in Table 1. There were no important clinical differences at baseline between patients completing the study and those who dropped out. The 2 groups of completing participants were balanced for baseline clinical variables (Table 1).

**Table 1:** Patient Demographics (VINDICATE) at Randomization: Intention-to-Treat Population

	Total (n = 163)	Placebo (n = 83)	Vitamin D (n = 80)
Male	129 (79.1)	62 (74.7)	67 (83.8)
Age, yrs	68.7 ± 13.10	69.0 ± 13.78	68.5 ± 12.45
Caucasian	146 (90.0)	74 (89.0)	72 (90.0)
Etiology			
Ischemic heart disease	94 (57.7)	50 (60.2)	44 (55.0)
Nonischemic cardiomyopathy	61 (37.4)	29 (34.9)	32 (40.0)
Valvular heart disease	8 (4.9)	4 (4.8)	4 (5.0)
Diabetes mellitus	37 (22.7)	20 (24.1)	17 (21.3)
BMI, kg/m^2^	30.0 ± 11.41	30.3 ± 14.36	29.8 ± 7.26
NYHA functional class			
II	145 (89.0)	71 (85.5)	74 (92.5)
III	18 (11.0)	12 (14.5)	6 (7.5)
Beta blockers	155 (95.1)	79 (95.2)	76 (95.0)
ACEi/ARB	150 (92.0)	76 (91.6)	74 (92.5)
Furosemide dose, mg/day	61.4 ± 46.38	64.4 ± 52.07	58.6 ± 41.00
Digoxin	29 (18.0)	15 (18.3)	14 (17.7)
Spironolactone	83 (51.2)	41 (50.0)	42 (52.5)
Device (ICD or CRT)	48 (29.5)	27 (32.5)	21 (26.3)
Atrial fibrillation,	68 (45.0)	33 (42.9)	35 (47.3)
Baseline heart rate, beats/min	70.5 ± 13.10	72.7 ± 14.72	68.2 ± 10.86
Systolic BP, mm Hg	120.3 ± 20.81	122.9 ± 22.44	117.6 ± 18.74
Diastolic BP, mm Hg	71.2 ± 13.21	72.8 ± 14.96	70.0 ± 10.99
6-min walk test, m	292.9 (120.35)	283.7 (116.84)	302.2 (123.81)
LVEF %	26.1 ± 10.68	26.5 ± 10.62	25.6 ± 10.80
LVEDD, mm	57.8 ± 7.58	58.0 ± 6.49	57.6 ± 8.62
LVESD, mm	50.3 ± 8.50	50.7 ± 7.58	49.8 ± 9.42
LVEDV, ml	163.0 ± 66.60	164.1 ± 60.07	161.8 ± 73.58
LVESV, ml	115.4 ± 59.39	119.4 ± 53.30	111.0 ± 63.58
25(OH) Vitamin D, nmol/l	37.3 ± 22.56	36.4 ± 20.24	38.2 ± 24.81
Parathyroid hormone, pmol/l	11.4 ± 8.09	11.7 ± 7.50	11.0 ± 8.75
Creatinine, μmol/l	96 ± 29.3	94.4 ± 29.42	96.6 ± 29.26

Values are n (%) or mean ± SD. Conversion factors: vitamin D nmol/l · 0.4 = ng/ml; creatinine mmol/l · 0.11 = mg/dl; calcium mmol/l · 4 = mg/dl; parathyroid hormone pmol/l · 9.4 = pg/ml.

ACEi = angiotensin-converting enzyme inhibitor; ARB = aldosterone receptor blocker; BMI = body mass index; BP = blood pressure; CRT = cardiac resynchronization therapy; ICD = implantable cardioverter defibrillator; LVEDD = left ventricular end-diastolic diameter; LVEDV = left ventricular end-diastolic volume; LVEF = left ventricular ejection fraction; LVESD = left ventricular end-systolic diameter; LVESV = left ventricular end-systolic volume; NYHA = New York Heart Association functional class; VINDICATE = VitamIN D treatIng patients with Chronic heArT failurE.

**Figure 2:**
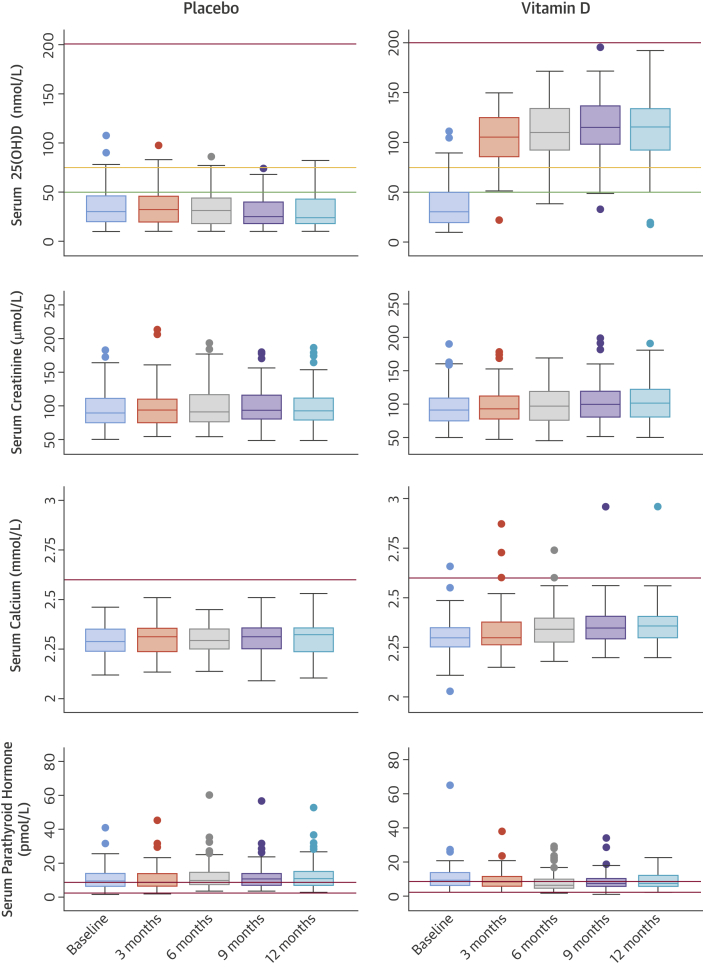
Median and Interquartile Ranges for Vitamin D, Creatinine, Calcium, and Parathyroid Concentrations at 3 Monthly Time Points in VINDICATE by Treatment Allocation Vitamin D concentrations are described in relation to deficiency **(green line),** sufficiency **(orange line)**, and the accepted upper limit for hypervitaminosis D **(red line)**. Serum calcium levels are described in relation to upper limit of normal range **(red line),** and serum parathyroid hormone concentrations in relation to the normal range **(between red lines)**. Conversion factors: vitamin D nmol/l · 0.4 = ng/ml; creatinine mmol/l · 0.11 = mg/dl; calcium mmol/l · 4 = mg/dl; parathyroid hormone pmol/l · 9.4 = pg/ml. VINDICATE = VitamIN D treatIng patients with Chronic heArT failurE.

The vitamin D_3_ supplement was well-tolerated and achieved sustained normal serum 25(OH)D concentrations by 3 months post-randomization, indicating excellent adherence to treatment (Figure 2). Patients in the placebo arm had lower median concentrations of 25(OH)D at 12-months post-randomization, (24.5 nmol/l; range: 10.0 to 81.8 nmol/l [9.8 ng/ml; range: 4 to 32.7 ng/ml]) than patients in the active supplement arm (115 nmol/l; range: 17.8 to 193 nmol/l [46 ng/ml; range: 7.1 to 77.2 ng/ml]; p < 0.0001) confirming the effectiveness of the vitamin D supplementation in normalizing vitamin D levels. The supplement also effectively normalized 1,25 (OH)_2_ vitamin D_3_ (calcitriol) levels to 121 pmol/l (range: 40 to 331 pmol/l [46.5 pg/ml; range: 15.4 to 127.3 pg/ml]) at 12 months and also suppressed PTH levels, leading to lower PTH levels in subjects allocated vitamin D (8.70 pmol/l; range: 1.28 to 22.2 pmol/l [82 ng/ml, range: 12 to 209 ng/ml]) than those allocated placebo (10.80 pmol/l; range: 2.80 to 53.10 pmol/l [102 ng/ml, range: 26 to 499 ng/ml]); analysis of covariance difference in mean change was -3.63 pmol/l, 95% CI: -5.24 to -2.03 pmol/l (-34 ng/ml, 95% CI: -49 to -19 ng/ml); p < 0.0001.

**Figure 3:**
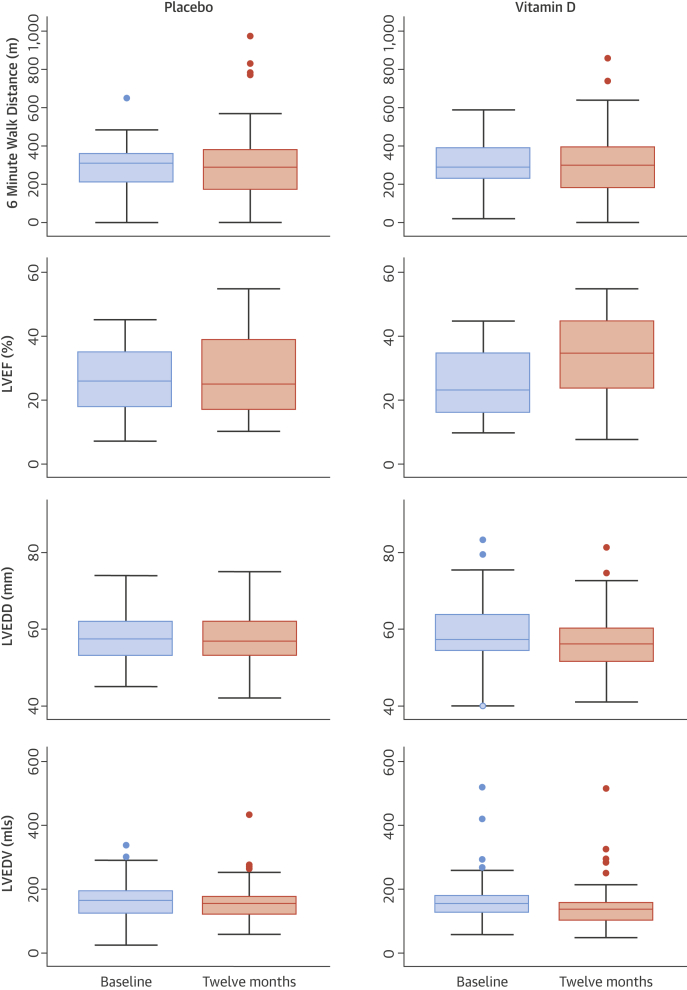
Median and Interquartile Ranges for 6-Minute Walk Test Distance, and LVEF, LVEDD, and LVEDV Measured by Echocardiography at Baseline and Final Visit in VINDICATE by Treatment Allocation LVEDD = left ventricular end-diastolic dimension; LVEDV = left ventricular end-diastolic volume; LVEF = left ventricular ejection fraction; VINDICATE = VitamIN D treatIng patients with Chronic heArT failurE.

No patient was observed to suffer hypervitaminosis D according to our pre-specified safety concentration of 200 nmol/l (80 ng/ml) 25(OH)D and no subject required a down-titration of dose. One patient with borderline hypercalcemia at baseline (2.66 mmol/l [10.64 mg/dl]) had persistent hypercalcemia throughout the study, and 1 other patient with hypercalcemia at 3 months (2.73 mmol/l [10.9 mg/dl]) had a normal calcium level by 6 months and throughout the remainder of the study (Figure 2). There was no concerning change in renal function (Figure 2) and there were no study drug-related admissions or adverse events. Twelve months of 4,000 IU of cholecalciferol did not improve or preserve 6MWT distance in chronic HF patients (Figure 3).

At 12 months, patients in the vitamin D arm had a greater improvement in echocardiographic measures of LV function compared with patients randomized to placebo. Changes in the treatment versus placebo arms were as follows: for LVEF +7.65% (95% CI: 5.21% to 10.09%) and +1.36% (95% CI: -0.38% to 3.11%), respectively (p < 0.0001); for LVEDD -2.45 mm (95% CI: -3.70 to -1.21 mm) and 0.08 mm (95% CI: -1.25 to 1.10 mm), respectively (p = 0.002); and for LVESD were -2.72 mm (95% CI: -4.52 to -0.92 mm) and -0.99 mm (95% CI: -2.31 to 0.33 mm), respectively (p = 0.043). Changes in LV volumes in the treatment versus the placebo arms were: LVEDV -16.47 ml (95% CI: -25.71 to -7.22 ml) and -3.83 ml (95% CI: -13.36 to 5.70 ml), respectively (p = 0.04); and LVESV -18.77 ml (95% CI: -25.96 to 9.59 ml) and -8.49 ml (95% CI: -17.98 to 1.01 ml), respectively (p = 0.041) (Table 2, Figure 3). There was a dose-response relationship between the increase in vitamin D levels and the increase in LVEF (coefficient 0.04; p = 0.023) and decrease in LVEDV (coefficient -0.02; p = 0.035).

**Table 2:** Change in Primary and Secondary Outcome Variables in VINDICATE at 12 Months Post-Randomization: Intention-to-Treat Population

Endpoint	Randomized Treatment	Mean Change After 12 Months	ANCOVA Difference in Mean Change	p Value
Primary outcome				
6-min walk distance, m	Placebo	10.10 (-20.77 to 40.96)	-24.11 (-65.81 to 17.60)	0.255
	Vitamin D	-12.56 (-40.80 to 15.68)		
Secondary outcomes				
LVEF, %	Placebo	1.36 (-0.38 to 3.11)	**6.07 (3.20 to 8.94)**	**<0.001**
	Vitamin D	7.65 (5.21 to 10.09)		
LVEDD, mm	Placebo	-0.08 (-1.25 to 1.10)	**-2.49 (-4.09 to -0.90)**	**0.002**
	Vitamin D	-2.45 (-3.70 to -1.21)		
LVESD, mm	Placebo	-0.99 (-2.31 to 0.33)	**-2.09 (-4.11 to -0.06)**	**0.043**
	Vitamin D	-2.72 (-4.52 to -0.92)		
LVEDV, ml	Placebo	-3.83 (-13.36 to 5.70)	**-13.11 (-25.63 to -0.60)**	**0.040**
	Vitamin D	-16.47 (-25.71 to -7.22)		
LVESV, ml	Placebo	-8.49 (-17.98 to 1.01)	**-12.65 (-24.76 to -0.54)**	**0.041**
	Vitamin D	-18.77 (-25.96 to -9.59)		

Values are mean change (95% confidence intervals); 95% significance shown in **bold**.

ANCOVA = analysis of covariance; other abbreviations as in Table 1.

Enrollment into VINDICATE did not mandate CMR imaging, and one-third of patients in VINDICATE had cardiac devices incompatible with CMR imaging. Only 69 patients volunteered to undergo baseline CMR scanning. The CMR data are further limited as a result of withdrawal or death during follow-up (n = 8), device implantation between baseline and follow-up (n = 2 implantable cardioverter defibrillators), patient refusal to undergo a second scan (n = 17), and technical problems with the second scan, such that we only had 34 patients with serial CMR images. Baseline characteristics of these patients are shown in Online Table 3. Patients agreeing to serial CMR scans were younger (61.5 years [range: 36.7 to 84.8 years] vs. 71.3 years [range: 28.1 to 92.3 years]; p < 0.0001) had better renal function (creatinine: 86 μmol/l [range: 43 to 114 μmol/l] vs. 102 μmol/l [range: 48 to 245 μmol/l]; p = 0.007) and were non-significantly less deficient in 25-(OH) vitamin D at baseline (43.9 nmol/l [range: 10.0 to 90.4 nmol/l] vs. 35.94 nmol/l [range: 10.0 to 111.0 nmol/l] or 17.6 ng/ml [range: 4.0 to 36.2 ng/ml] vs. 14.4 ng/ml [range: 4.0 to 44.4 ng/ml]; p = 0.07), but were otherwise similar to patients who declined CMR scanning including the change in vitamin D from baseline to completion (p = 0.64). The data from serial CMR scans showed improvements in cardiac function with vitamin D, but were not statistically significant possibly due to insufficient statistical power: for LVEF 4.12% (95% CI: -0.11% to 8.35%) versus 1.19% (95% CI: -3.20% to 5.59%; p = 0.317), for LVEDV -26.12 ml (95% CI: -63.27 to 11.04 ml) versus –0.10 ml (95% CI: -12.88 to 13.07 ml; p = 0.168) and for LV -29.61 ml (95% CI: -72.40 to 13.18 ml) versus –1.36 ml (95% CI: -19.19 to 16.48 ml; p = 0.206). There was however, a dose response relationship in our CMR data, with a relationship between increases in vitamin D and reductions in LVEDV (coefficient -0.19; p = 0.050) and LVESV (coefficient -0.20; p = 0.083).

## Discussion

VINDICATE aimed to examine the effect of high-dose vitamin D_3_ supplementation in patients with chronic HF secondary to LVSD who underwent optimal medical therapy. The results demonstrate that 4,000 IU vitamin D_3_ given for 12 months is safe, well tolerated, and not associated with concerning adverse biochemical effects (Central Illustration).

**Central Illustration:**
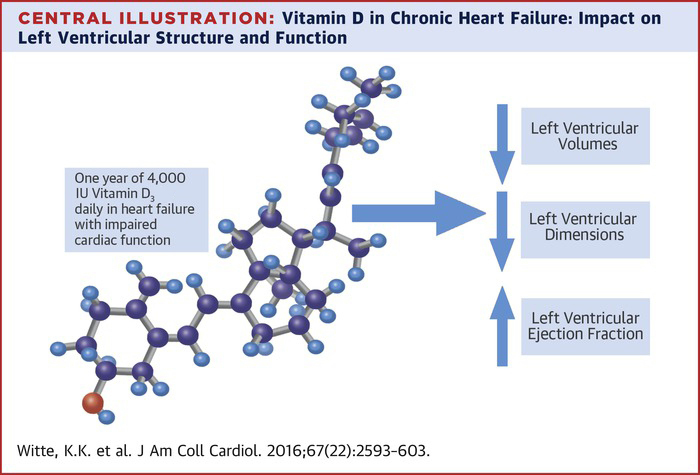
Vitamin D in Chronic Heart Failure: Impact on Left Ventricular Structure and Function Vitamin D improves left ventricular ejection fractions and reduces left ventricular dimensions and volumes.

There was no effect of vitamin D supplementation on the primary endpoint of 6MWT distance but there were statistically significant and prognostically and clinically relevant improvements in the secondary outcomes of LVEF and LV dimensions and volumes, thus suggesting that vitamin D is leading to beneficial reverse remodeling.

New therapies for serious chronic conditions including chronic HF are often expensive, increasingly technical, and frequently fail to meet the rigorous demands of large phase 3 clinical trials. Vitamin D might be an inexpensive and safe additional option for chronic HF patients and may have beneficial effects on multiple features of the syndrome (13).

Patients with chronic HF are frequently deficient in vitamin D, and low vitamin D levels increase the risk of incident chronic HF (26), and are associated with more severe disease and worse outcomes in patients with chronic HF 6, 7, 8, 9, 12. Supplementation to treat or prevent osteoporotic fractures might be associated with a lower incidence of chronic HF (10).

However, despite the publication of studies exploring various doses and forms of vitamin D supplementation in patients with chronic HF, there remains considerable uncertainty regarding the benefits of this therapeutic approach. In the first study by Schleithoff et al. (15), 93 subjects received 50 μg vitamin D_3_ + calcium (Ca^2+^) per day for 9 months or placebo + Ca^2+^. There was a trend to improvement of LV function measured by echocardiography and a smaller increase in pro-inflammatory cytokines during follow-up in those randomized to vitamin D. Both groups were given Ca^2+^ and both groups had some improvement in LV function with no differences between them. Witham et al. (16) examined vitamin D_2_ supplementation in 105 elderly patients. Subjects were randomized to 2 doses of 100,000 IU of vitamin D_2_ or placebo at baseline and 10 weeks and assessed at 20 weeks. There was no effect on walk distance or immune function, and a slight deterioration in quality of life. The population in that study was heterogeneous; patients with and without LVSD were included, mean N-terminal B-type natriuretic peptide levels and daily furosemide doses were lower than those seen in a usual HF population, medical therapy was not optimized, the duration of treatment was short, patients who were randomized to vitamin D remained deficient (43.4 nmol/l [17.4 ng/ml]), and PTH was not suppressed (27). Although Boxer et al. 28, 29 did not demonstrate improvements in cardiac function or objective measures of muscle strength and exercise capacity in 64 chronic HF patients (of whom 34 underwent echocardiography) randomized to weekly doses of 50,000 IU of vitamin D_3_ for 6 months, there was an improvement in serum aldosterone and quality of life in those allocated the supplement. In an open-label study, Schroten et al. (30) demonstrated a reduction in plasma renin concentration after 6 weeks of 2,000 IU vitamin D_3_ daily in 101 patients with chronic HF. Finally, Dalbeni et al. (31) noted an increase in LVEF of almost 7% after only 25 weeks in 13 patients randomized to 600,000 IU vitamin D_3_ at baseline and 2 further doses of 100,000 IU at 10 weeks and 20 weeks, whereas 10 patients randomized to placebo had a reduction in LVEF of more than 4%. The authors did not comment on cardiac dimensions and there was an increase in natriuretic peptide levels in both groups. In contrast to these studies, VINDICATE is a double-blind, placebo-controlled study of an oral non−calcium-based daily supplement of 4,000 IU of vitamin D_3_ administered for 12 months in patients with chronic HF due to LVSD on otherwise optimal medical therapy. The supplement led to consistent biochemical evidence of replenishment and an effective suppression of PTH levels.

The primary endpoint of VINDICATE was change in 6MWT distance. The study was based upon pilot data and powered to detect a 28-m difference between the 2 groups at 12 months (19). The variability in the walk distance measure at baseline was much greater than predicted from our pilot study, such that our sample size only had 7% post hoc power to detect a difference between the groups. VINDICATE was therefore underpowered to detect a clinically relevant change in walk distance. Six-minute walk distance is an increasingly frequently used patient-oriented outcome measure, but has greater variability than objective surrogate endpoints (20). The findings from VINDICATE have implications for future studies using 6-min walk distance as an outcome measure.

However, our secondary endpoints of cardiac function and structure measured by echocardiography were highly statistically and clinically significant, with improvements in LVEF, and LV dimensions and volumes. Similar changes were seen in a subgroup of patients agreeing to serial CMR imaging, although they did not reach conventional levels of statistical significance due to lack of power.

A pathophysiological hallmark of chronic HF secondary to LVSD is a progressive increase in LV cavity dimensions and impaired contractility, a process known as LV remodeling (32). Current accepted therapies for chronic HF which afford HF patients improvements in survival such as angiotensin converting enzyme inhibitors (33), beta-adrenoceptor antagonists 34, 35, and cardiac resynchronization therapy (36) have also been shown to have a favorable effect on LV remodeling by delaying progression of, or reversing LV dilatation. The degree of favorable remodeling induced by these treatments is related to long-term outcomes (37). It is therefore plausible that the improvements in cardiac function demonstrated in VINDICATE have the potential to improve outcomes.

### How does vitamin D contribute to beneficial remodeling?

Vitamin D deficiency could contribute to adverse remodeling through 2 major pathways. Vitamin D deficiency could lead to cardiomyocyte dysfunction by interfering with Ca^2+^ transport (38) at a cellular concentration. HF is a condition of intracellular calcium overload that adversely affects both contraction and relaxation. Furthermore, vitamin D deficiency might contribute to cardiomyocyte hypertrophy, interstitial inflammation, and fibrosis (39). Hence, vitamin D deficiency could contribute to a more rapid progression to HF following myocardial damage due to more aggressive adverse remodeling (40).

However, adverse remodeling is also the result of persistent neurohormonal activation, particularly that of the renin angiotensin aldosterone system (RAAS) which strongly contributes to deteriorating cardiac function, cardiomyocyte loss, and interstitial fibrosis (41). Inhibition of the RAAS leads to attenuated or reverse LV remodeling in patients with HF (42). Vitamin D deficiency heightens RAAS activity 30, 43, whereas vitamin D supplementation seems to reduce renin synthesis (44) and plasma renin activity (43).

### Study limitations

VINDICATE was performed at a single center. However, the study was based upon results from a randomized, placebo-controlled pilot study in 53 patients using the same dose for 12 months that also showed a favorable effect of vitamin D on cardiac structure and function (19). We did not examine the effect of vitamin D supplementation in patients with chronic HF and preserved ejection fraction, a group of patients who may warrant such investigation.

## Conclusions

VINDICATE has demonstrated that high-dose vitamin D_3_ supplementation is safe, well-tolerated, and associated with a clinically relevant improvement in cardiac function in chronic HF patients already taking current optimal therapies.

Perspectives **COMPETENCY IN PATIENT CARE AND PROCEDURAL SKILLS:** In patients with chronic HF, vitamin D deficiency is common, and high-dose vitamin D_3_ supplementation is safe, well tolerated, and associated with a favorable effect on cardiac function.

**TRANSLATIONAL OUTLOOK:** Further studies are needed to establish the mechanism by which correction of vitamin D_3_ deficiency improves cardiac function in patients with systolic HF and the generalizability of this response.
