# Supplementary material for: Effects of Vitamin D on Cardiac Function in Patients With Chronic HF: The VINDICATE Study
Source: J Am Coll Cardiol. 2016 Jun 7;67(22):2593–603. doi: 10.1016/j.jacc.2016.03.508 (PMC4893154; doi:10.1016/j.jacc.2016.03.508)

**Supplementary Table 1: Patient Demographics at Randomization in Proof of Concept Study: Intention-to-Treat Population**

|  | **Placebo (n=26)** | **Vitamin D (n=27)** | **Total (n=53)** |
| --- | --- | --- | --- |
| **Male Sex (n)[%]** | 20 (76.9%) | 23 (85.2%) | 43 (81.1%) |
| **Age** | 68.9 (10.50) | 74.1 (7.98) | 71.6 (9.59) |
| **Etiology (n)[%]** |  |  |  |
| Dilated cardiomyopathy | 6 (23.1%) | 8 (29.6%) | 14 (26.4%) |
| Ischemic heart disease | 19 (73.1%) | 19 (70.4%) | 38 (71.7%) |
| Valvular heart disease | 1 (3.8%) | 0 (0.0%) | 1 (1.9%) |
| **Diabetes mellitus (n)[%]** | 4 (15.4%) | 5 (18.5%) | 9 (17.0%) |
| **NYHA (n)[%]** |  |  |  |
| II | 21 (80.8%) | 18 (65.7%) | 39 (73.6%) |
| III | 5 (19.2%) | 9 (33.3%) | 14 (26.4%) |
| **Beta blockers (n)[%]** | 22 (84.6%) | 27 (100.0%) | 49 (92.5%) |
| **ACEi/ARB (n)[%]** | 25 (96.2%) | 24 (88.9%) | 49 (92.5%) |
| **Furosemide dose (mg/day)** | 53.1 (35.30) | 48.9 (38.56) | 50.9 (36.70) |
| **Digoxin (n)[%]** | 7 (26.9%) | 7 (25.9%) | 14 (26.4%) |
| **Spironolactone (n)[%]** | 11 (42.3%) | 13 (48.1%) | 24 (45.3%) |
| **Atrial fibrillation (n)[%]** | 10 (38.5%) | 13 (48.1%) | 23 (43.4%) |
| **Systolic BP (mmHg)** | 127.2 (22.03) | 119.3 (19.93) | 123.2 (21.16) |
| **Diastolic BP (mmHg)** | 70.8 (10.80) | 65.9 (14.76) | 68.3 (13.09) |
| **LVEF (%)** | 34.0 (7.36) | 32.2 (6.95) | 33.1 (7.14) |
| **LVEDD (mm)** | 58 (11) | 59 (8.6) | 59 (9.8) |
| **LVESD (mm)** | 48 (13.3) | 49 (8.7) | 49 (11.1) |
| **LVEDV (mls)** | 156.0 (67.46) | 166.3 (72.41) | 161.3 (69.55) |
| **LVESV (mls)** | 105.5 (53.05) | 114.0 (57.39) | 109.8 (54.94) |
| **Vitamin D (nmol/L)** | 31.0 (17.37) | 24.4 (9.63) | 27.4 (13.99) |
| **Parathyroid hormone (pmol/L)** | 9.6 (5.27) | 10.6 (5.02) | 10.1 (5.11) |

Continuous variables are mean (SD), categorical variables are n (%) as indicated.

NYHA, New York Heart Association class; ACEi; angiotensin converting enzyme inhibitor, ARB; aldosterone receptor blocker, BP; blood pressure, LVEF; left ventricular ejection fraction; LVEDD, left ventricular end-diastolic diameter; LVESD, left ventricular end-systolic diameter; LVEDV, left ventricular end-diastolic volume; LVESV, left ventricular end-systolic volume.

**Supplementary table 2: Changes in echocardiographic variables with vitamin D supplementation adjusted for baseline for all patients in the intention-to-treat population of the proof of concept study**

| **Endpoint** | **Randomized treatment** | **Mean  (adjusted for baseline) 95% CI** | **Mean difference in change (adjusted for baseline)** | **95% CI** | **p-value** |
| --- | --- | --- | --- | --- | --- |
| LVEF (%) | Placebo | 35.8 [33.16, 38.51] | 2.7 | [-1.01, 6.43] | 0.1490 |
|  | Vitamin D | 38.5 [35.98, 41.11] | . |  | . |
| LVEDD (mm) | Placebo | 57 [ 55.4, 59.1] | -1.0 | [-3.4, 1.7] | 0.5212 |
|  | Vitamin D | 56 [ 54.7, 58.2] | **.** |  | **.** |
| LVESD (mm) | Placebo | 47 [ 44.6, 50.0] | **-4.0** | **[-7.6, -0.1]** | **0.0428** |
|  | Vitamin D | 43 [ 40.9, 46.0] | **.** |  | **.** |
| LVEDV (mls) | Placebo | 177.0 [163.0, 191.1] | **-23.4** | **[-42.86, -3.94]** | **0.0196** |
|  | Vitamin D | 153.6 [140.2, 167.1] | **.** |  | **.** |
| LVESV (mls) | Placebo | 116.1 [104.5, 127.6] | **-19.5** | **[-35.44, -3.49]** | **0.0181** |
|  | Vitamin D | 96.6 [85.56, 107.6] | . |  | . |

LVEF, left ventricular ejection fraction; LVEDD, left ventricular end-diastolic diameter; LVESD, left ventricular end-systolic diameter; LVEDV, left ventricular end-diastolic volume; LVESV, left ventricular end-systolic volume.

**Supplementary table 3: Baseline variables of patients undergoing serial CMR scans - intention-to-treat population**

|  | **Placebo (n=17)** | **Vitamin D (n=17)** | **Total (n=34)** |
| --- | --- | --- | --- |
| **Male Sex (n)[%]** | 12 [71] | 14 [82] | 26 [76] |
| **Age** | 60.6 (16.0) | 62.3 (16.8) | 61.4 (16.2) |
| **Etiology (n)[%]** |  |  |  |
| Ischemic heart disease | 14 [41] | 6 [35] | 20 [59] |
| Non-ischemic cardiomyopathy | 3 [18] | 11 [65] | 13 [38] |
| **Diabetes mellitus (n)[%]** | 4 [24] | 1 [6] | 5 [15] |
| **BMI** | 27.6 (3.8) | 26.8 (5.7) | 27.2 (4.8) |
| **NYHA (n)[%]** |  |  |  |
| II | 17 [100] | 17 [100] | 34 [100] |
| **Beta blockers (n)[%]** | 16 [94] | 15 [88] | 31 [91] |
| **ACEi/ARB (n)[%]** | 17 [100] | 17 [100] | 34 [100] |
| **Furosemide dose (mg/day)** | 33.5 (61.7) | 34.1 (40.0) | 33.8 (51.2) |
| **Digoxin (n)[%]** | 0 [0] | 2 [12] | 2 |
| **Spironolactone (n)[%]** | 9 [53] | 8 [47] | 17 [50] |
| **Atrial fibrillation (n)[%]** | 2 [12] | 8 [47] | 10 [29] |
| **Heart rate (beats/min)** | 68.8 (11.1) | 68.8 (9.6) | 68.8 (10.2) |
| **Systolic BP (mmHg)** | 119.5 (17.5) | 107.0 (19.3) | 113.3 (19.3) |
| **Diastolic BP (mmHg)** | 70.7 (11.3) | 67.8 (9.6) | 69.3 (10.4) |
| **CMR-LVEF (%)** | 33.6 (8.0) | 38.2 (12.1) | 36.0 (10.4) |
| **CMR-LVEDV (mls)** | 214.2 (61.2) | 211.8 (105.6) | 213.0 (85.0) |
| **CMR-LVESV (mls)** | 144.5 (55.0) | 139.5 (106.1) | 142.0 (83.1) |
| **Creatinine (μmol/L)** | 88.1 (28.6) | 83.3 (16.2) | 85.7 (23.0) |
| **Vitamin D (nmol/L)** | 43.0 (22.8) | 44.6 (34.3) | 43.9 (23.2) |
| **Parathyroid hormone (pmol/L)** | 8.7 (4.3) | 8.1 (3.9) | 8.4 (4.0) |

Continuous variables are mean (SD), categorical variables are n (%) as indicated.

NYHA, New York Heart Association class; ACEi; angiotensin converting enzyme inhibitor, ARB; aldosterone receptor blocker, BP; blood pressure, LVEF; left ventricular ejection fraction; LVEDD, left ventricular end-diastolic diameter; LVESD, left ventricular end-systolic diameter; LVEDV, left ventricular end-diastolic volume; LVESV, left ventricular end-systolic volume.

Supplementary Figure 1: Consort diagram for the randomized, placebo-controlled, proof of concept study of vitamin D_3_ supplementation.


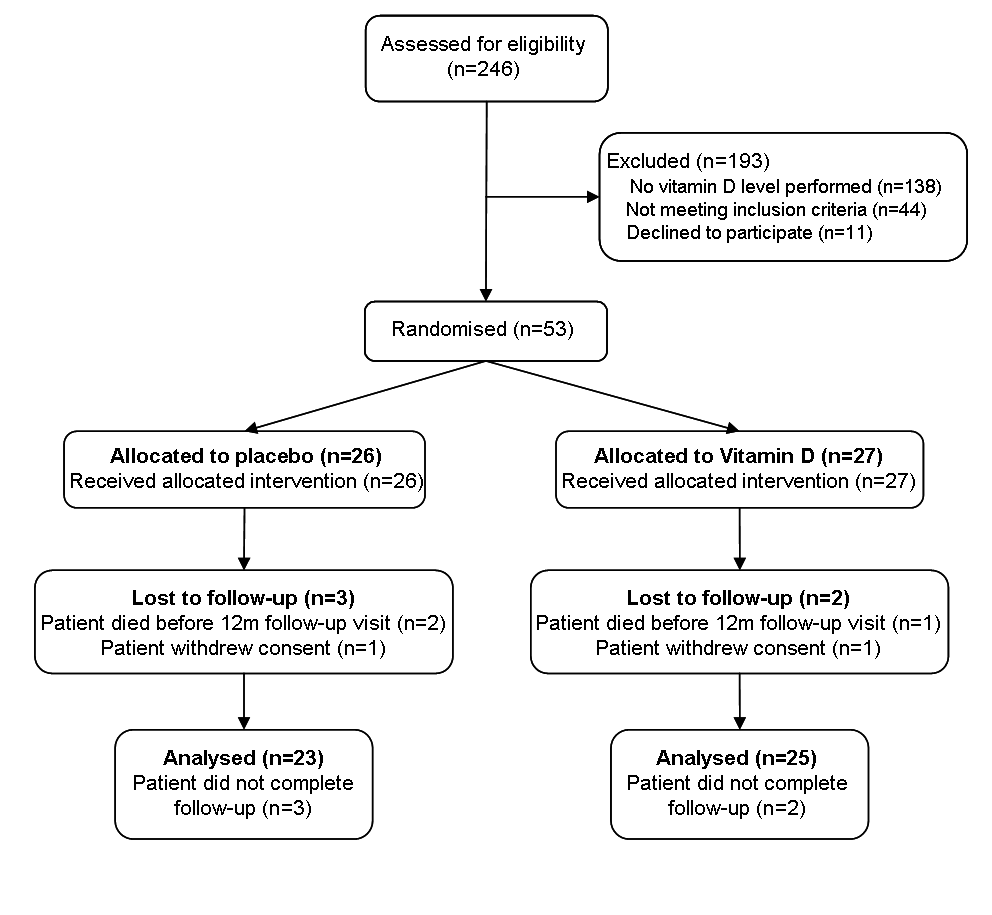


Supplementary figure 2: Biochemical changes during the randomized, placebo-controlled, proof of concept study of vitamin D_3_ supplementation by allocated group. Vitamin D concentrations are described in relation to deficiency (green line), sufficiency (yellow) and the accepted upper limit for hypervitaminosis D (red line). Serum calcium levels described in relation to upper limit of normal range (red line), and serum PTH concentrations in relation to the normal range (between red lines).


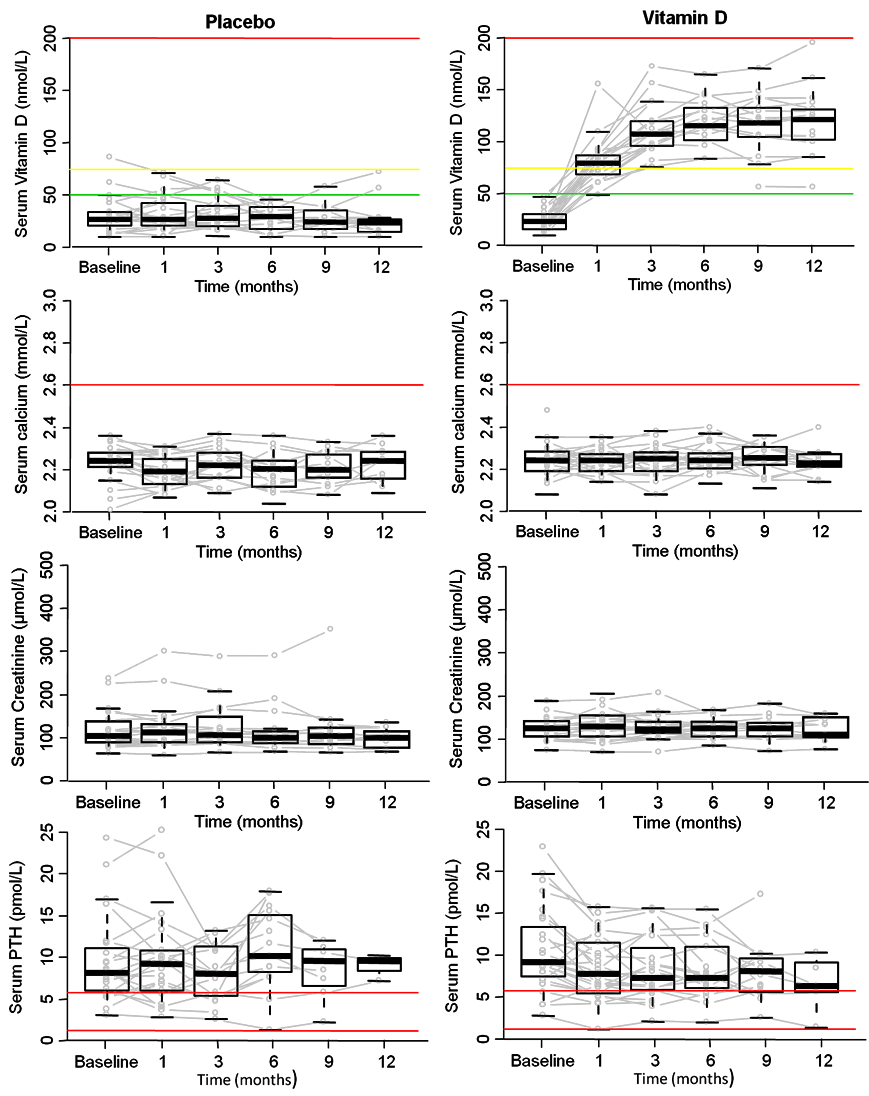

Supplement: Online Tables 1–3 and Online Figures 1 and 2 [file mmc1.doc]
